# Pyorrhœa Alveolaris: A Case in Practice

**Published:** 1904-09

**Authors:** H. C. Fitzhardinge


### PYORRHŒA ALVEOLARIS: A CASE IN PRACTICE.

BY H. C. FITZHARDINGE, D.D.S.

Miss R., aged twenty-nine, a stenographer by occupation, presented for treatment during the latter part of November, 1903. On examining her mouth I found that she was wearing a full upper plate. She said all her upper teeth had become loose and had been extracted. The gum had receded from her lower six anterior teeth, which were very loose. The teeth had a dense, opaque appearance, the gum was puffy, of a bluish livid hue, and had a shiny, polished appearance on the surface. Pus was exuding from pockets around the teeth. I diagnosed this as a case of so-called pyorrhœa alveolaris. Considering the age of the patient, and also the fact that she was most anxious to have these teeth saved if possible, the prognosis was favorable. I removed the accretions of calculus from the roots very carefully, washed out the pockets with hydrogen dioxide, cauterized the necrosed tissue with fifty per cent. sulphuric acid, washed out with a stream of warm water, and followed with the Truman method. With the exception of the sulphuric acid, this treatment was continued once a week (the patient could not come oftener) until April, 1904, using the sulphuric acid about every second visit and reducing the strength to twenty-five per cent. I prescribed a mouth-wash, No. 1 (see below), to be used three times daily, and she is now using the mouth-wash No. 2, as it is a little more palatable, to be continued all the time. The teeth are now firm and the gums, though receded, are healthy.

No. 1.

Hydronaphthol, gr. xv;
Sp. vini rect.,
Aquæ dest., āā ℥ii. M.

Sig.—Two to ten drops in a glass of water. Use with a brush as a mouth-wash.

No. 2.

℞ Hydronaphthol, ʒii;

Ol. cinnamomi, ♏xv;

Ol. menth. pip.,

Ol. gaultheriæ, āā ♏v;

Sp. vini rect., ℥iiss;

Aq. dest., ℥iv. M.

Sig.—Twenty drops to a tumbler of water twice daily with a brush as a mouth-wash.

